# Intravenous Immunoglobulin in Acute Exacerbations of Fibrotic Interstitial Lung Diseases: A Retrospective, Real-World Study

**DOI:** 10.3390/medicina61091594

**Published:** 2025-09-04

**Authors:** Vasilina Sotiropoulou, Eva Theochari, Matthaios Katsaras, Panagiota Tsiri, Dimitrios Komninos, Ioannis Christopoulos, Georgios Tsirikos, Christina Kalogeropoulou, Dimitrios Daoussis, Kyriakos Karkoulias, Fotios Sampsonas, Argyrios Tzouvelekis

**Affiliations:** 1 Department of Respiratory Medicine, University Hospital of Patras, 26500 Patras, Greeceatzouvelekis@upatras.gr (A.T.); 2 Department of Radiology, University Hospital of Patras, 26500 Patras, Greece; 3 Department of Rheumatology, University Hospital of Patras, 26500 Patras, Greece

**Keywords:** intravenous immunoglobulin, acute exacerbation, interstitial lung diseases, idiopathic pulmonary fibrosis

## Abstract

*Background and Objectives*: Despite the devastating impact of acute exacerbations of fibrotic interstitial lung diseases (AE-fILDs), established treatment strategies are majorly lacking. The therapeutic potential of intravenous immunoglobulin (IVIG) in AE-fILDs was explored considering its anti-inflammatory and immunomodulatory effects, as well as the immunocompromised status of fILD patients and the high frequency of infections that AE-fILDs triggers. *Materials and Methods*: This was an observational, retrospective study. We investigated the therapeutic potential of IVIG in patients hospitalized for AE-fILDs between May 2021 and November 2024. *Results*: We included 39 patients diagnosed with AE-fILDs. All patients received IVIG (total dose of 1 g/kg, divided into three daily doses), pulse corticosteroids for three days and broad-spectrum antibiotics. No adverse events were considered to be related to IVIG therapy during the study period. The in-hospital and the 90-day mortality were 10 (26%) and 13 (33%) patients, respectively. Twenty-nine patients (74%) were discharged and 18 of them (62%) were in need of long-term oxygen therapy. The mean PaO_2_/FiO_2_ ratio (P/F ratio) was 183 mmHg on admission and 294 mmHg on discharge (*t*-test, *p* < 0.0001). *Conclusions*: This study suggests a potential therapeutic signal, indicating that IVIG is a relatively harmless, well-tolerated, and a potentially effective add-on treatment to current therapeutic approaches. Further research is essential to clarify the role of IVIG, determine optimal treatment protocols, and assess its efficacy in different ILD subtypes.

## 1. Introduction

The management of fibrosing interstitial lung diseases (ILDs) still presents with significant challenges, mainly due to the unpredictable clinical course and the lack of disease-modifying agents. Approximately one third of patients will develop a progressive fibrotic phenotype, with gradual clinical, radiological and functional deterioration; yet episodes of acute respiratory decompensation may complicate the disease course at any point [1]. Acute exacerbation of fibrotic interstitial lung diseases (AE-fILDs) is defined as an acute worsening of respiratory status in patients with a previous or concurrent ILD diagnosis. It is characterized by marked respiratory symptoms and the appearance of new bilateral alveolar abnormalities on chest computed tomography typically of less than one month duration, which cannot be fully explained by fluid overload [2,3]. Although most commonly described in idiopathic pulmonary fibrosis (IPF), exacerbations have also been reported in other fILDs, such as idiopathic nonspecific interstitial pneumonia (NSIP), connective tissue disease-associated ILDs (CTD-ILDs), and fibrotic hypersensitivity pneumonitis (HP), with a lower incidence compared to IPF [4]. AE-ILDs can even occur in patients with preserved lung volumes and mild imaging abnormalities or with previously undiagnosed fibrotic ILDs [2,3,5].

The diagnosis is based on clinical and radiological findings [2,6]. Triggering Factors may include infections, gastroesophageal reflux disease, drugs, air pollution, aspiration and in some cases perioperative lung injury [5,7]. Even though the pathogenetic mechanisms underlying AE-ILDs have not been fully elucidated, inflammation and immunity play a pivotal role. Elevated levels of pro-inflammatory cytokines, high cellularity of the bronchoalveolar lavage fluid, and histopathological findings of diffuse alveolar damage are highly suggestive of an aberrant inflammatory response interplayed with exuberant alveolar cell apoptosis. Inflammatory changes may extend beyond the lungs with systemic consequences and multi-organ dysfunction [8,9,10]. Moreover, recent evidence highlights the role of antibodies in the pathogenesis of AE-ILDs through various mechanisms, including autoimmunity, complement activation, and direct cytotoxicity [11,12,13].

AE-ILDs are associated with poor prognosis, significant morbidity, and mortality. The in-hospital and short-term mortality for acute exacerbation of IPF(AE-IPF) may exceed 50%, rising to 75–90% in intubated patients. Other ILDs generally have a better prognosis, though post-exacerbation mortality varies widely [4,14,15]. Despite the devastating impact of AE-fILDs, established treatment strategies are majorly lacking. The latest IPF guidelines propose the use of corticosteroids, despite limited supporting evidence [1]. Some retrospective studies indicate that corticosteroids may provide benefits in AE-IPF and other fILDs [2,3,16]; yet results are still conflicting [17,18]. Importantly, as seen in a recently published global survey, the majority of physicians across the world apply pulses of methylprednisolone in AE-IPF [15]. Other immunosuppressants, such as rituximab, cyclophosphamide, and azathioprine, are frequently used, although their efficacy remains uncertain [16]. Additionally, considering the potential risk for typical or opportunistic infections, broad-spectrum antibiotics are often administered empirically [2,3]. Finally, concerning the use of antifibrotic agents in AE-IPF, evidence indicates that both anti-fibrotics exert a rather preventive rather than therapeutic role considering that nintedanib may prolong the time to the first AE-IPF, while pirfenidone could lower the risk of respiratory-related hospital admissions [19,20,21,22]. Based on the recent survey, for patients not previously treated with antifibrotics, physicians generally initiated such therapy after clinical stabilization. Conversely, for patients already on antifibrotic therapy at the time of AE-IPF, the majority of physicians continued the treatment [15].

Intravenous immunoglobulin (IVIG) is a biologic component of pooled antibodies from healthy donors’ plasma. IVIG is used as a replacement treatment in immunodeficiencies, a hyperimmune treatment targeting specific infectious agents and in hematological, neurological, and rheumatological diseases, given its immunomodulatory and anti-inflammatory properties [23]. Interestingly, IVIG is also used in some cases of rapidly progressive ILDs other than IPF, particularly in myositis-associated ILDs [24]. IVIG therapy has been linked to both immediate and delayed adverse events. Immediate reactions typically occur within 30–60 min after infusion initiation. These are often transient and mild, such as headache, low-grade fever, or fatigue. Moderate manifestations, including nausea, vomiting, or chest discomfort, may also arise. Such reactions can usually be alleviated through symptomatic management, premedication with anti-inflammatory agents or antihistamines, reduction in infusion rate, dose adjustment, or discontinuation of therapy. More serious complications, though less frequent, include thromboembolic phenomena, hemolytic episodes, renal dysfunction, arrhythmias, marked fever, hemodynamic instability, dyspnea, or fluid overload. [25,26].

In light of the evidence that infections represent major causes of AE-fILDs [14,27], that patients with fILDs exhibit a state of immunosuppression, affecting both cellular and humoral immunity [28], and that dysregulated inflammatory and immunological processes are major contributors in AE-fILDs [10], we hypothesized that there is a profound rationale to investigate the safety and efficacy of IVIG treatment in AE-fILDs.

## 2. Materials and Methods

**Figure 1:**
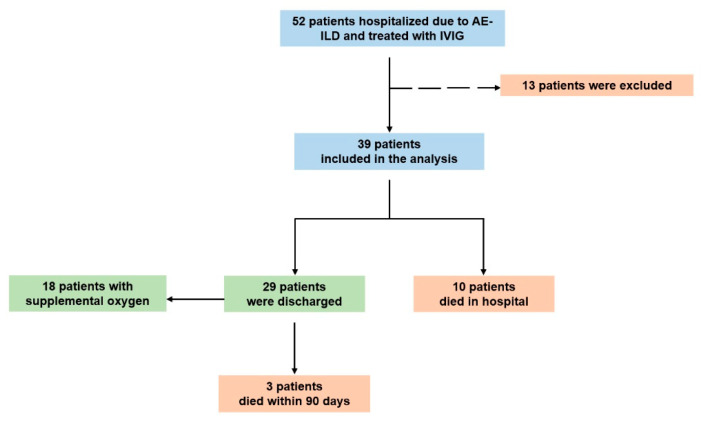
Study flowchart. Abbreviations: AE-fILD: acute exacerbation of fibrotic interstitial lung disease, IVIG: intravenous immunoglobulin.

In this investigator-initiated, retrospective, observational study, we enrolled consecutive patients with AE-fILDs treated with IVIG between May 2021 and November 2024 (Figure 1). The trial was conducted at the University Hospital of Patras, a referral ILD center in Greece. Retrospective data collection and analysis was approved by our institutional review board and local ethics committee (4758-13/02/25). Each patient provided a written informed consent to participate in our institutional standard-of-care protocol for AE-fILDs management, including corticosteroids, antibiotics, prophylactic anticoagulation, and IVIG, as further analyzed below. The institutional standard-of-care protocol was informed on the basis of the limited published evidence and the available clinical practice expertise regarding the management of AE-fILDs. This protocol was designed to guide the treatment of a highly challenging condition, while allowing individualized therapeutic decisions. IVIG was incorporated as an optional intervention within this framework, despite the limited supporting evidence. For the purposes of the present study, we specifically analyzed only those patients who received IVIG.

Fibrotic ILDs were diagnosed based on the established criteria, after integration of the clinical and imaging findings in a multidisciplinary discussion [1,29,30]. The diagnosis of AE-ILDs was reached according to the International Working Group Report of Acute Exacerbation of IPF criteria, which were also applied to the other fibrotic ILDs, through a multidisciplinary discussion [1,2]. Upon admission, all patients underwent computed tomography pulmonary angiogram and cardiac ultrasound, to exclude pulmonary embolism and fluid overload, respectively. Patients with decompensated cardiac failure, newly diagnosed pulmonary embolism, severe acute respiratory syndrome coronavirus-2 infection, and comorbidities indicating administration of IVIG, such as myasthenia gravis and chronic lymphocytic leukemia, were excluded from the analysis (Figure 1).

Upon hospitalization, patients promptly underwent comprehensive diagnostic evaluations, including routine laboratory tests, blood, urine and sputum cultures, and multiplex viral panel analysis, to identify potential infectious triggers. In patients deemed clinically stable and fit for the procedure, bronchoscopy was performed to obtain BAL samples for microbiological culture and detailed analysis. Frequent laboratory assessments were performed throughout patients’ hospital stay to ensure close monitoring and early detection of potential disease- or treatment-related complications.

All patients included in this study received corticosteroids, empiric antimicrobial therapy, and IVIG. More specifically, corticosteroid therapy consisted of intravenous methylprednisolone pulses at 500–1000 mg/day for three consecutive days, followed by methylprednisolone at 1 mg/kg with gradual tapering. Antacids were co-administered, and trimethoprim–sulfamethoxazole prophylaxis was given when indicated. Broad-spectrum antibiotics were given empirically, most commonly macrolides or fluoroquinolones, or alternatively piperacillin–tazobactam and linezolid at standard dosing. When pathogens were identified, antimicrobial therapy was adjusted according to the antibiogram. The total IVIG dose was 1 g/kg body weight, divided into three consecutive daily infusions. As recommended [26], the infusion was initiated at 0.5–1 mL/kg/hour for the first 15–30 min, and in the absence of adverse reactions, the rate was progressively increased every 15–30 min to a maximum of 3–6 mL/kg/hour. Patients were continuously monitored during infusion, and in cases of mild or moderate adverse events, symptomatic management and reduction in the infusion rate were applied. Depending on the underlying diagnosis, clinical condition, and response to initial therapy, some patients additionally received immunosuppressive agents, such as rituximab at a dose of 500 to 1000 mg followed by a second administration of 500 to 1000 mg two weeks later, and intravenous cyclophosphamide at a dose of 500 to 1000 mg. Treatment with antifibrotic agents, nintedanib or pirfenidone, was maintained in the absence of contraindications.

Clinical, laboratory, and imaging data and outcomes were retrospectively extracted from hospital medical records and outpatient follow-up notes. Collected variables included demographic information (age, sex), underlying or newly diagnosed ILD, comorbidities, HRCT imaging patterns, baseline therapies, in-hospital treatment regimens including administered oxygen therapy, laboratory parameters such as IVIG, C-reactive protein, and procalcitonin levels at admission, and bronchoscopy findings. Pulmonary function measurements obtained at discharge were also collected. Clinical outcomes—including length of hospitalization, in-hospital mortality and 90-day survival—were recorded. The PaO_2_/FiO_2_ ratio (P/F ratio) was calculated on admission and at discharge, when arterial blood gas measurements were available.

IVIG safety was evaluated by retrospectively recording major adverse events occurring during or after infusion, including thromboembolic events, hemolysis, renal dysfunction, arrhythmias, significant fever, hemodynamic instability, and fluid overload. Clinical outcomes and the PaO_2_/FiO_2_ ratio were evaluated as indirect measures of IVIG efficacy. Additionally, a subgroup analysis was conducted in patients who received rituximab in combination with standard therapy. Statistical analyses were performed using MedCalc statistical software (version 20.104). Normality of distribution was examined using the Kolmogorov–Smirnov test. Continuous variables following normal distribution are presented as mean ± SD, otherwise as median (95% confidence interval, CI). Patients’ characteristics on admission and discharge were compared using paired samples *t*-test. Mortality rates in the rituximab and non-rituximab subgroups were compared using Fisher’s exact test. Differences were considered statistically significant at *p* < 0.05.

## 3. Results

### 3.1. Baseline Characteristics

This study included 39 patients suffering from AE-fILDs. Patients’ baseline characteristics are presented in Table 1. There were 19 (49%) males, 15 (38%) never-smokers, with mean ± SD age 70 ± 12 years. A total of 12 patients (31%) had a diagnosis of IPF, 11 (28%) of connective tissues disease associated-ILDs (CTD-ILDs), 5 (13%) of idiopathic fibrotic non-specific interstitial pneumonia, and 3 (8%) of fibrotic hypersensitivity pneumonitis. Six patients (15%) fulfilled the progressive pulmonary fibrosis criteria before AE-fILDs. Interestingly, ILD diagnosis was not previously established in eight (21%) patients. A total of 16 patients (41%) were on antifibrotics—nintedanib: 9 patients (23%), pirfenidone: 7 patients (18%)—and 6 patients (15%) were under long-term oxygen therapy (LTOT) before hospitalization. Ten patients (26%) received mycophenolate mofetil and seven patients (18%) received low doses (≤4 mg) of methylprednisolone per os at baseline. No patients had received rituximab or cyclophosphamide prior to study enrolment.

The mean ± SD IgG levels on admission were 998 ± 447 mg/dL, within normal limits. Due to the patients’ critical condition, bronchoscopy was feasible in 12 patients (31%) and specific pathogens were identified in only 3 cases (Table 2). All patients received IVIG (total dose of 1 g/kg, divided in three consecutive daily doses), pulse corticosteroids (methylprednisolone 500–1000 mg/day for three days), and broad-spectrum antibiotics. A total of 25 patients (64%) also received other immunosuppressants during hospitalization for AE-fILD—rituximab: 23 patients (59%), cyclophosphamide: 2 patients (5%). Antifibrotics were not discontinued during hospitalization, if not otherwise contraindicated. The majority of patients (64%) were supported with high-flow nasal cannula, six (15%) with continuous positive airway pressure, and eight (21%) were intubated. The therapeutic interventions for AE-fILDs are summarized in Table 2.

### 3.2. IVIG Exerts an Acceptable Safety Profile in Patients with AE-fILDs

No major adverse events were considered to be related to IVIG therapy during the study period. While minor infusion-related reactions cannot be entirely excluded, no clinically significant complications such as thromboembolic events, hemolysis, renal dysfunction, arrhythmias, significant fever, hemodynamic instability, and fluid overload were noted in our cohort, suggesting that IVIG was generally well tolerated in this patient population.

### 3.3. IVIG Was Associated with Favorable Clinical Outcomes in Patients with AE-fILDs

**Figure 2:**
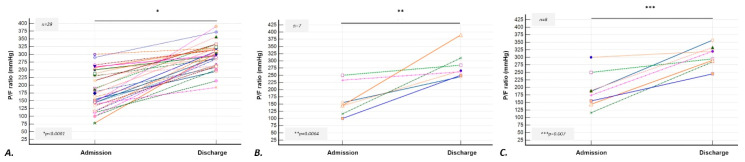
PaO_2_/FiO_2_ ratio change on admission and on discharge—Panel (**A**) In patients with AE-fILDs (n = 29)—Mean P/F ratio ± SD on admission 183 ± 63 mmHg VS on discharge 294 ± 43 mmHg, *p* < 0.0001 (Paired samples *t*-test). Panel (**B**) In patients with AE-IPF (n = 7)—Mean P/F ratio ± SD on admission 163 ± 57 mmHg VS on discharge 287 ± 51 mmHg, *p* = 0.0064 (Paired samples *t*-test). Panel (**C**) In patients with AE-CTD-ILDs (n = 8)—Mean P/F ratio ± SD on admission 190 ± 59 mmHg VS on discharge 306 ± 34 mmHg, *p* = 0.007 (Paired samples *t*-test). Abbreviations: AE-fILD: acute exacerbation of fibrotic interstitial lung disease, CTD-ILDs: connective tissue disease associated interstitial lung disease, IPF: Idiopathic pulmonary fibrosis, IVIG: intravenous immunoglobulin, P/F ratio: PaO_2_/FiO_2_ ratio, SD: standard deviation.

The mean ± SD duration of hospitalization was 12 ± 2 days. The in-hospital and the 90-day mortality were 10 (26%) and 13 (33%) patients, respectively. A total of 29 patients (74%) were discharged and 18 of them (62%) were in need of LTOT (Figure 1). Among the available data, six patients (21%) experienced a recurrent episode of AE-fILD within one-year post-discharge. The mean PaO_2_/FiO_2_ ratio (P/F ratio) was 183 mmHg on admission and 294 mmHg on discharge (*t*-test, *p* < 0.0001) (Figure 2—Panel A). The mean forced vital capacity (%predicted) was 66%, and the mean diffusing capacity for carbon monoxide (%predicted) was 49% on discharge. Moreover, in IPF subgroup, the in-hospitality mortality was five patients (42%) and the mean P/F ratio of the survivors was 163 mmHg on admission and 286 mmHg on discharge (*t*-test, *p* = 0.0064) (Figure 2—Panel B). In CTD-ILDs subgroup, the in-hospital mortality was three patients (27%) and the mean P/F ratio of the survivors was 190 mmHg on admission and 306 mmHg on discharge (*t*-test, *p* = 0.007) (Figure 2—Panel C).

### 3.4. Subgroup Analysis in Rituximab and Non-Rituximab Group

Moreover, an analysis comparing patients who received corticosteroids, IVIG, and rituximab versus those who received only corticosteroids and IVIG was performed. In-hospital mortality was eight patients (35%) in the rituximab group and two patients (14%) in the non-rituximab group (fourteen patients), though the difference was not statistically significant (Fisher’s exact test, *p* = 0.26). Similarly, three-month mortality was nine patients (39%) in the rituximab group and four patients (29%) in the non-rituximab group, with no statistically significant difference (Fisher’s exact test, *p* = 0.72).

## 4. Discussion

Το the best of our knowledge, our study represents one of the few published studies addressing real-world safety and efficacy profiles of IVIG administration in patients suffering from AE-fILDs. The rationale supporting this study was based on its anti-inflammatory and immunomodulatory effects, as well as the immunocompromised status of fILD patients and the high frequency of infections that AE-fILDs trigger. We have demonstrated that IVIG exerts an acceptable safety profile in patients with AE-fILDs and was associated with favorable clinical outcomes, as indicated by rates of discharge and improved oxygenation.

In this retrospective analysis, IVIG administration was not associated with any major adverse events. While minor infusion-related reactions cannot be completely ruled out, no serious complications—including thromboembolic events, hemolysis, renal impairment, arrhythmias, significant fever, hemodynamic instability, or fluid overload—were observed in our cohort. These observations suggest that IVIG is generally well-tolerated in patients with AE-fILDs. By comparison, other immunosuppressive or cytotoxic therapies commonly used in this context, such as cyclophosphamide and rituximab, carry a higher risk of severe toxicity. Nevertheless, further studies are needed to more comprehensively evaluate IVIG safety profile in AE-fILDs.

Our findings are in line with other previously published studies. In particular, previous retrospective underpowered studies also demonstrated positive clinical outcomes in patients with AE of fibrotic idiopathic interstitial pneumonias under IVIG treatment as indicated by a significantly higher 90-day survival than controls [31]. A prospective study to further investigate the effect of IVIG on AE-IPF in Japan (jRCT1061220010) is currently pending. Moreover, Kulkarni et al. showed that the combination of therapeutic plasma exchange, rituximab, and IVIG may result in decreased supplemental oxygen needs in patients with AE-IPF. An ongoing randomized, multicenter trial—STRIVE-IPF (NCT03286556)—aims to compare the efficacy of triple combined autoantibody reduction therapy versus usual care in AE-IPF, with six-month survival as the primary endpoint [32]. One possible explanation is that IVIG functions similarly to plasma exchange, leading to the depletion of autoantibodies harmful to lung epithelium—particularly in AE of CTD-ILDs. Alternatively, IVIG may enhance humoral immunity against potential infectious agents responsible for AE-fILDs.

AE-fILDs are associated with a high burden of disease, including need for LTOT, and increased mortality, particularly during hospitalization and the immediate post-discharge period [3,14]. In our cohort, mortality during hospitalization and 90-days post discharge was 26% and 33%. Prior studies indicate one-month survival after AE-fILDs of 66% and three-month survival of 44%, while in-hospital mortality after AE-IPF often exceeds 50% [15,27]. Similarly, post-exacerbation mortality in ILDs has been reported to range from 33% to 83% [3]. Despite that our findings suggest a comparatively lower mortality compared to that observed in previous studies, this difference may reflect variations in baseline patient characteristics, disease severity, and management approaches, and should be interpreted cautiously with regard to a positive therapeutic impact. Moreover, patients’ P/F ratio on admission showed significant improvement by discharge, and a substantial minority of discharged patients did not even require LTOT. Nevertheless, nearly two thirds of discharged patients remained dependent on LTOT. This observation aligns with previous studies, which reported persistent hypoxemia in approximately 61% of AE-IPF survivors, and indicated that 40–60% of patients with fILDs without baseline respiratory failure may require LTOT following an acute exacerbation [33,34].

Over the years, several AE-fILD therapeutic approaches have shown promise in retrospective studies but failed to demonstrate efficacy in prospective trials. Beyond the hypothesis that the anticoagulant effects of thrombomodulin-alfa could improve outcomes in AE-IPF, the randomized trial did not show a 90-day survival benefit [35]. Additionally, despite a significant proportion of pulmonologists reporting the use of cyclophosphamide for AE-IPF [15], a phase three trial showed that adding cyclophosphamide to glucocorticoids increased 90-day mortality [17]. Therefore, results should be interpreted with caution until additional studies provide further validation.

We acknowledge that our study has several limitations. Its retrospective nature, small sample size, absence of a comparator arm, and adjustment for confounding factors do not allow rigid conclusions on drug efficacy and restrict the generalizability of our findings. Furthermore, the co-administration of IVIG with other therapies, such as rituximab, may have influenced the outcomes. In-hospital and 90-day mortality did not differ significantly between patients who received rituximab and those who received only corticosteroids and IVIG. Given the small sample size, the analysis may lack sufficient power to detect a true difference, if one exists. These findings should be confirmed through a prospective, controlled study with careful adjustment for confounding factors for more reliable conclusions to be drawn. Despite these limitations, our study contributes meaningful clinical observations and real-world experience in managing AE-fILDs, an area where evidence remains scarce.

## 5. Conclusions

In conclusion, as stated in the manuscript, our study can by no means provide evidence-based support for the efficacy of IVIG, given the limitations discussed above. However, our data provide preliminary evidence of a potential benefit, indicating that IVIG is a relatively harmless (less toxic than other immunosuppressants or cytotoxic agents proven harmful in both randomized controlled trials and retrospective studies), well-tolerated, and a potentially effective add-on treatment to current therapeutic approaches, either corticosteroid- or non-corticosteroid based. Further research and well-designed randomized controlled trials are essential to clarify the role of IVIG, determine optimal treatment protocols, and assess its efficacy in different ILD subtypes. We hope that our findings will contribute to these efforts and help pave the way for more targeted and effective therapeutic strategies for this life-threatening condition.

## Figures and Tables

**Table 1:** Characteristics of patients at baseline.

Baseline Characteristics	(N, %)
Age (years) (Mean ± SD)	70 ± 12
Male/Female	19 (49%)/20 (51%)
Comorbidities	
Dyslipidemia	26 (67%)
Arterial hypertension	22 (56%)
Diabetes mellitus	14 (36%)
Coronary artery disease	11 (28%)
Depression	11 (28%)
Thyroid disorder	6 (15%)
Chronic obstructive pulmonary disease	5 (13%)
Underlying ILD diagnosis	
Idiopathic pulmonary disease	12 (31%)
Connective tissues disease associated ILDs	11 (28%)
[RA/Myositis/MPA/SLE]	[5 (45%)/3 (28%)/2 (18%)/1 (9%)]
Idiopathic fibrotic NSIP	5 (13%)
Fibrotic hypersensitivity pneumonitis	3 (8%)
Newly diagnosed ILD	8 (21%)
Underlying CT patterns (28 available)	
Definite/Probable/Indeterminate UIP	6 (21%)/5 (18%)/5 (18%)
Fibrotic NSIP	10 (36%)
Fibrotic hypersensitivity pneumonitis	2 (7%)
Prior treatment	
Antifibrotics: nintedanib/pirfenidone	9 (23%)/7 (18%)
Corticosteroids (≤4 mg methylprednisolone/day)	7 (18%)

Abbreviations: CT: Computed tomography, ILD: interstitial lung disease, IVIG: intravenous immunoglobulin, MPA: Microscopic polyangiitis, NSIP: Non-specific interstitial pneumonia, RA: Rheumatoid arthritis, SD: standard deviation, SLE: Systemic lupus erythematosus, UIP: Usual interstitial pneumonia.

**Table 2:** AE-fILDs investigation and management.

Investigation and Treatment	(N, %)
Laboratory exams	
IVIG on admission (mg/dL) (Mean ± SD)	998 ± 447
CRP on admission (mg/dL) (Mean ± SD)	11.57 ± 8.3
PCT on admission (μg/L) (Median, 95%CI)	0.07 (0.05 to 0.13)
Bronchoscopy	12 (31%)
Pharmacological treatment	
IVIG	39 (100%)
Methylprednisolone pulse therapy	39 (100%)
Empiric antibiotics	39 (100%)
Anticoagulation	39 (100%)
Rixutimab/Cyclophosphamide	23 (59%)/2 (5%)
Supportive oxygen therapy	
High flow nasal cannula	25 (64%)
Continuous positive airway pressure	6 (15%)
Invasive mechanical ventilation	8 (21%)

Abbreviations: CI: confidence interval, CRP: C-reactive protein, IVIG: intravenous immunoglobulin, SD: standard deviation, PCT: procalcitonin.

## Data Availability

The original contributions presented in this study are included in the article. Further inquiries can be directed to the corresponding author.

## Abbreviations

The following abbreviations are used in this manuscript:

AE-fILDs	acute exacerbation of fibrotic interstitial lung diseases
AE-IPF	acute exacerbation of idiopathic pulmonary fibrosis
BAL	bronchoalveolar lavage
CI	confidence interval
CT	computed tomography
CTD-ILDs	connective tissue disease-associated interstitial lung diseases
CRP	C-reactive protein
fILDs	fibrotic interstitial lung diseases
ILDs	interstitial lung diseases
IPF	idiopathic pulmonary fibrosis
IVIG	intravenous immunoglobulin
LTOT	long-term oxygen therapy
MPA	microscopic polyangiitis
NSIP	non-specific interstitial pneumonia
PCT	procalcitonin
P/F ratio	PaO_2_/FiO_2_ ratio
RA	rheumatoid arthritis
SD	standard deviation
SLE	systemic lupus erythematosus
UIP	usual interstitial pneumonia
